# A Moderated Mediation Analysis of Condom Negotiation and Sexual Orientation on the Relationship Between Sexual Coercion and Condom Use in Chinese Young Women: Cross-Sectional Study

**DOI:** 10.2196/24269

**Published:** 2021-01-19

**Authors:** Wen Zhang, Edmond Pui Hang Choi, Daniel Yee-Tak Fong, Janet Yuen-Ha Wong

**Affiliations:** 1 School of Nursing Li Ka Shing Faculty of Medicine University of Hong Kong Hong Kong Hong Kong

**Keywords:** sex offenses, sex orientation, unprotected sex, online research, women’s health

## Abstract

**Background:**

The high prevalence of sexual coercion against young women has become a significant public health issue in China and other regions around the world. Young women are also especially vulnerable to engage in inconsistent condom use because of low sexual control. Although the relationship between sexual coercion and condom use has been widely demonstrated, the mechanism of this relationship is still unclear.

**Objective:**

The objective of this study was to test condom negotiation as a mediator of the relationship between sexual coercion and condom use in young Chinese women and to investigate whether sexual orientation is a moderator.

**Methods:**

Data were collected using web-based questionnaires and a total of 402 young Chinese women were included in the analysis. Sexual coercion was measured using a subscale of the Revised Conflict Tactics Scales and condom negotiation was measured using a subscale of the UCLA Multidimensional Condom Attitudes Scale. Sexual orientation was assessed using an item adopted from a previous study and condom use was calculated by the total number of times condoms were used divided by the total number of times sexual intercourse was engaged in during the past 3 months. Moderated mediation analyses were conducted with sexual coercion as the independent variable, condom use consistency as the dependent variable, condom negotiation as the mediator variable, and sexual orientation as a moderator.

**Results:**

The moderated mediation analysis indicated that the relationship between sexual coercion and condom use was significantly mediated by condom negotiation and moderated by sexual orientation. The indirect effect of condom negotiation was significant in heterosexual women (indirect effect: –0.80, 95% boot CI –1.67 to –0.36) but not in sexual minority women (indirect effect: –0.33, 95% boot CI –0.86 to 0.31).

**Conclusions:**

The results showed that sexual orientation meaningfully affects the relationship between sexual coercion and condom negotiation. The difference in the mechanism of the relation between sexual coercion and sexual behaviors in heterosexual and sexual minority women should be considered for future research and interventions aimed at mitigating the adverse effects of sexual coercion.

## Introduction

Sexual coercion against women remains a significant global health problem [1]. Previous studies have defined sexual coercion as behaviors, ranging from verbal manipulation to physical force, employed to complete or attempt sexual activities without the partner’s free consent [2,3]. A national survey in the United States found that approximately one-fifth of women reported experiencing sexual violence in their lifetime; one-half of these women reported that intimate partners were the offenders [4]. This national survey further indicated that more than 1 in every 3 female survivors of rape was first raped in her college-aged years (18-24 years) [4]. According to research by Planty et al [5], the risk of sexual coercion was higher in the age group of 18 to 34 years than in other age groups.

Sexual coercion against young women in China has also become an emerging public health issue that deserves attention [6]. The prevalence of sexual coercion against Chinese college women was approximately 13% in 2008 [7], and a similar prevalence was found in 2015, despite the improved status of women in Hong Kong, China [8]. Young women are also especially vulnerable to inconsistent condom use, since low sexual control has consistently been reported in young Chinese women [9]. A national survey in China reported that 1.6% of Chinese female college students had multiple sexual partners [10] and another study reported that only 17.2% of sexually active college women in China consistently used condoms [11]. An association between sexual coercion and inconsistent condom use has been observed [12,13]. Most of the data indicated that individuals with a history of sexual coercion (versus those without) reported a higher level of inconsistent condom use, which resulted in a higher risk of contracting sexually transmitted infections (STIs) [14]. Although previous studies have explored the mechanism of the relation between a history of sexual violence and condom use in female sex workers [15] and HIV-positive women [16], the specific mechanism of the relation between a history of sexual coercion and condom use in college women remains unclear. To improve interventions aimed at reducing sexual risk among college women, it is necessary to understand the mechanism underlying the relation between sexual coercion and condom use in this group.

Condom negotiation is one of the strongest predictors of condom use [17]. A previous study indicated that condom negotiation might play a crucial role in the relation between sexual violence and condom use [15]. Condom negotiation is closely related to condom use by college women [18], and women with a history of sexual coercion are less likely to negotiate or use condoms than women without experiences of sexual coercion. According to the traumagenic dynamics model [19,20], sexual coercion is viewed as a traumatic event with psychological sequelae, such as a negative attitude arising from the powerlessness experienced during sexual coercion. This negative attitude then contributes to maladaptive behavioral patterns. Women with an abusive experience could be at a disadvantage in their condom negotiations with their sexual partners because they seek to avoid nonphysical coercion [21]. Thus, they may be more likely to have limited or no control over condom decision making, which contributes to inconsistent condom use. Taken together, these patterns suggest that condom negotiation is a potential mediator between sexual coercion and condom use.

Previous studies found that sexual minority women (women who identify as having a sexual orientation other than heterosexual or who engage in same-sex sexual behavior, experience same-sex attraction, or self-identify as lesbian or bisexual [22]) experience a significantly higher incidence of sexual coercion than heterosexual women [23]. This suggests that although the experience of sexual coercion has a disincentivizing effect on the consistency of condom use, it does not affect all women equally. Sexual orientation also plays a role in individuals’ condom use and the negotiation process. Young bisexual women exhibited a greater likelihood of inconsistent condom use in vaginal intercourse than heterosexual women [24]. Skakoon-Sparling and Cramer [25] found that the process of condom negotiation can be impacted by sexual orientation. This finding might suggest that personal characteristics such as sexual orientation moderate the association between the experience of sexual coercion and consistency of condom use.

**Figure 1:**
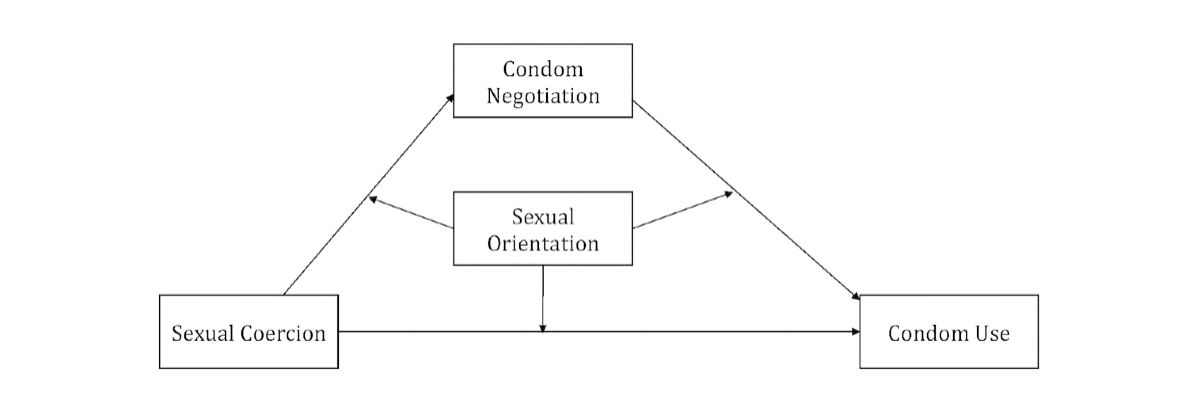
Proposed moderated mediation model.

In this study, we explored the relationship between sexual coercion and condom use in a sample of Chinese college women. Based on previous literature, we investigated the mediating effect of condom negotiation on the relation between sexual coercion and condom use in this population. Unique to this study, we tested whether sexual orientation moderated the hypothesized mediation of the relation between the experience of sexual coercion and condom use by condom negotiation. The proposed moderated mediation model is shown in Figure 1.

## Methods

### Data Collection

The baseline data of an interactive computer-based intervention (ICBI) project [26] were used to perform the mediated moderation analysis. This project was a randomized controlled trial that estimated the relative effectiveness of an ICBI and the provision of basic information in terms of promoting consistent condom use. The baseline data were collected from September 2018 to December 2018. The protocol for the parent study was approved by Institutional Review Boards (IRB NO.UW-17029) and is registered at ClinicalTrials.gov (NCT03695679). Signed informed consent was obtained from each participant via the study’s website. Data were collected via an anonymous web-based survey conducted at 5 universities in Hong Kong SAR, China. Vouchers of HK $300 (US$38.69) were delivered to participants who completed the project; participants who only completed the baseline questionnaire did not receive vouchers.

### Participants and Procedures

We recruited female students from 5 universities in Hong Kong by bulk email using the corresponding institution’s bulk email delivery service. In addition, we displayed posters on the campuses and set up campus booths to distribute leaflets. Students who were interested in the study were asked to complete a registration form with their contact information via a Google Form. The invitation email with the website registration information was sent to all interested students, and they were then screened after they logged into the website. Participants who met the inclusion criteria were asked to provide informed consent; they then completed a baseline assessment. Participants could not submit the questionnaire if any information was missing. The inclusion criteria in this study were female college students who were aged 18 years or older, were unmarried, reported having intimate partners in the past 12 months, and had engaged in sexual activity in the past 3 months. Women were excluded if they were unwilling to complete the questionnaire, were pregnant or had recently given birth, or had psychiatric illness. We screened 1503 students, of whom 805 did not meet the eligibility criteria and 292 refused to participate. Of the 406 eligible participants, 4 participants were excluded after data checking (3 participants reported having no sexual experience but also reported engaging in sexual activity in the past 3 months and 1 participant had a missing sexual coercion scale because of a technical problem), giving a validity rate of 99.0%. Ultimately, 402 female university students were included in the study. The average age of the participants was 21.90 (SD 2.74) years and the average age at first sexual intercourse was 19.48 (SD=2.40) years. Among the 402 participants, 87.6% (n=352) had never smoked, 10.7% (n=43) were quitting smoking, and 1.7% (n=7) were smokers; 33.3% (n=134) never drank, 16.4% (n=66) were quitting drinking, and 50.2% (n=202) drank. Approximately 70.6% (284/402) of the participants were born in Hong Kong and 29.4% (118/402) were born elsewhere.

### Measures

Sexual coercion was measured using a 7-item subscale of the Revised Conflict Tactics Scales [27]. Participants rated items to indicate how often the behavior occurred during the past year on a 7-point Likert scale, where higher scores indicated higher frequency. This scale has been widely used in the Chinese population and has shown a satisfactory reliability [28]. In this study, Cronbach was 0.63 for this subscale.

Sexual orientation was assessed using an item that was adopted from the longitudinal Growing Up Today Study [29], which had been ongoing since 1996. There were 6 options: completely heterosexual (attracted only to the opposite sex), mostly heterosexual, bisexual (attracted to both the opposite and the same sex), mostly homosexual, completely homosexual (attracted only to the same sex), and unsure. Referring to the definition of sexual minority women [22] and a previous study in the Chinese population [30], completely heterosexual was coded as “heterosexual,” and mostly heterosexual, bisexual, mostly homosexual, completely homosexual, and unsure were combined into a “sexual minority” group.

Condom use was measured by the consistency of condom use, which was defined as the total number of times condoms were used during vaginal intercourse divided by the total number of times vaginal intercourse occurred in the past 3 months. This assessment was recommended by a systematic review of condom use measurement that examined 56 studies of sexual risk behavior [31].

Condom negotiation was measured using a subscale of the UCLA Multidimensional Condom Attitudes Scale [32]. This subscale is used to evaluate attitudes toward condom negotiation and use (eg, “When I suggest using a condom, I am almost always embarrassed,” “I am comfortable talking about condoms with my partner,” “I never know what to say when my partner and I need to talk about condoms or other protection,” and “It is easy to suggest to my partner that we use a condom”). These items were answered using a 7-point Likert scale from “strongly agree” to “strongly disagree.” Higher scores indicate a more positive attitude regarding communication and negotiation of condom use. A previous study has shown acceptable validity and reliability in the Chinese population [33]. In this study, Cronbach was 0.87.

Demographic variables examined in the study included participant characteristics such as age, age at first sexual intercourse, smoking status, drinking status, and place of birth.

### Statistical Analyses

Descriptive statistics and bivariate correlation analyses were conducted of the studied variables as preliminary analyses. The Shapiro-Wilk test was used as a test of normality and *P*<.05 was considered evidence for nonnormality. For skewed data, the median and IQR were used to describe the nonnormal variables, the Mann-Whitney U test was used to test the difference between the heterosexual group and the sexual minority group, and Spearman rank correlation analyses were conducted to identify the correlations between the nonnormal variables. The mediation effect of condom negotiation was tested using model 4 of Hayes’ PROCESS macro for SPSS (version 25.0; IBM Corp) [34]. Moderated mediation analysis was conducted using model 59 of PROCESS to examine whether the indirect path was moderated by sexual orientation [34]. Since the data on the consistency of condom use may be nonnormally distributed, a bootstrapping procedure with 5000 samples was used to test the proposed conditional direct and indirect effects using the PROCESS macro for SPSS. Age and age at first sexual intercourse were added as covariates.

## Results

### Normality and Description of the Study Variables

The results of the Shapiro-Wilk test showed that condom use (*P*<.001), sexual coercion (*P*<.001), and condom negotiation (*P*<.001) were not normally distributed. The descriptive statistics and differences in the study variables between the heterosexual group and the sexual minority group are presented in Table 1. The results of the Mann-Whitney U test indicated that there was a significant difference in condom use (U=7841, Z=–6.23, *P*<.001) and condom negotiation (U=10740.5, Z=–2.43, *P*=.02), between the heterosexual group and the sexual minority group. Further, Spearman rank correlation analyses showed that in the heterosexual group, those who had more frequent sexual coercion experiences reported significantly less condom use (r_s_=–0.36, *P*<.001) and were less positive about condom negotiation (r_s_=–0.28, *P*<.001); there was a significant positive correlation between condom use and condom negotiation (r_s_=0.30, *P*<.001). In the sexual minority group, only condom use was positively related to condom negotiation (r_s_=0.24, *P*=.03).

**Table 1:** Description of the study variables and results of the Mann-Whitney U test.

Variables	Sexual orientation		Mann-Whitney U test		
	Heterosexual (n=321), median (IQR)	Sexual minority (n=81), median (IQR)	U	Z	*P* value
Sexual coercion	0 (2)	0 (0)	12428.5	–0.76	.45
Condom use	100 (33.33)	0 (100)	7841	–6.23	<.001
Condom negotiation	30 (9)	28 (9.5)	10740.5	–2.43	.02

### Tests of Mediation

The results of the mediation analysis regarding sexual coercion and condom use, after adjustments for age and age at first sexual intercourse, showed that an experience of sexual coercion was a negative predictor of condom negotiation (coefficient a=–0.16, 95% boot CI –0.31 to –0.10) (Table 2), indicating that participants who experienced sexual coercion were less likely to engage in condom negotiation. Condom negotiation was a positive predictor of condom use (coefficient b=2.02, 95% boot CI 1.32 to 2.70), which indicated that participants who were more positive about condom negotiation were more likely to be consistent in terms of condom use. A significant indirect and negative effect of sexual coercion on the consistency of condom use through condom negotiation was found (indirect effect: coefficient a=–0.32, 95% boot CI –0.67 to –0.18). The direct effect of sexual coercion on condom use became nonsignificant (coefficient c=–0.33, 95% boot CI –0.86 to 0.31). The indirect effect accounted for 49.2% of the total effect of sexual coercion on condom use.

**Table 2:** Mediation results for condom negotiation.

Outcome, Predictor		Coefficient	Boot SE	Boot LLCI^a^	Boot ULCI^b^
**Condom negotiation**					
	Sexual coercion	–0.16	0.06	–0.31	–0.10
**Condom use**					
	Sexual coercion	–0.33	0.29	–0.86	0.31
	Condom negotiation	2.02	0.35	1.32	2.70
Direct effect		–0.33	0.29	–0.86	0.31
Indirect effect		–0.32	0.13	–0.67	–0.18
Total effect		–0.65	0.28	–1.25	–0.12

^a^LLCI: lower limit confidence interval.

^b^ULCI: upper limit confidence interval.

### Tests of Moderated Mediation

**Figure 2:**
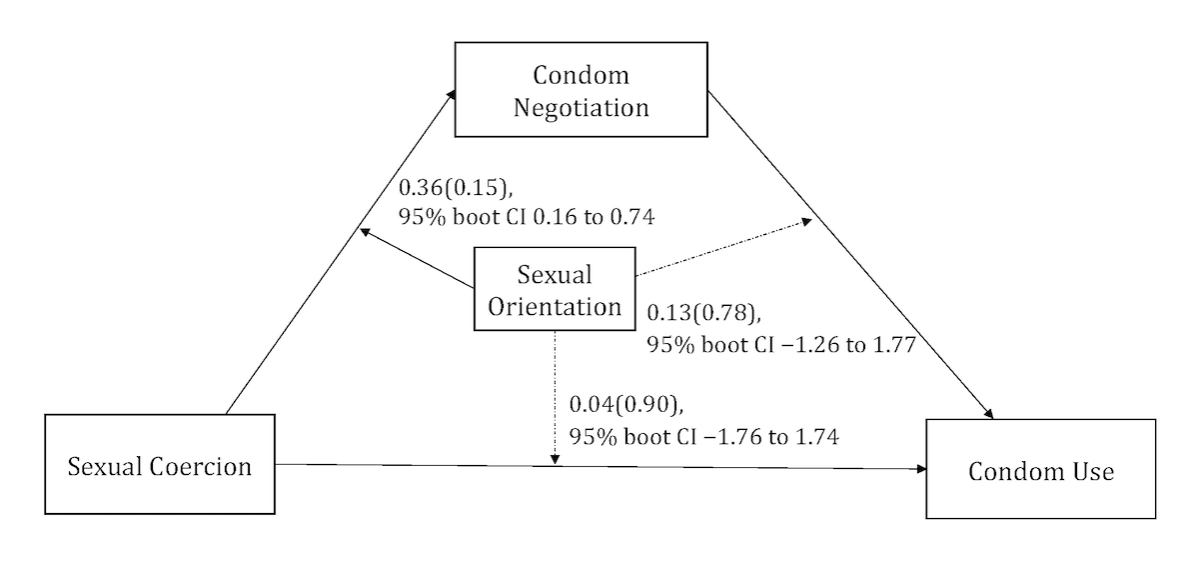
Tested moderated mediation model.

**Figure 3:**
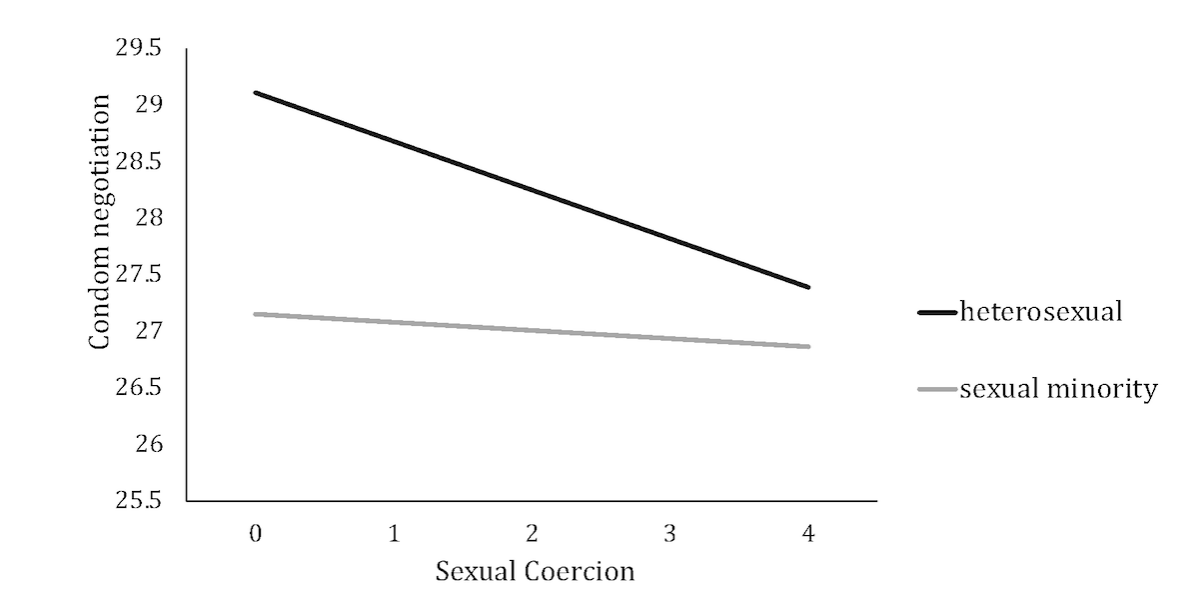
The moderation of the relationship between sexual coercion and condom negotiation by sexual orientation.

After adjusting for age and age at first sexual intercourse, the results of the moderated mediation analyses for sexual coercion and condom use showed that the interaction term between sexual coercion and sexual orientation was significant (coefficient c= 0.36, 95% boot CI 0.16 to 0.74) (Table 3 and Figure 2), which suggested that sexual orientation moderated the association between sexual coercion and condom negotiation. To further explore the moderation effect, the conditional indirect effect of sexual coercion on condom use via condom negotiation was estimated by using the pick-a-point approach in both sexual orientation groups. A significant indirect effect was seen in the heterosexual group (effect = –0.80, 95% boot CI –1.67 to –0.36), while the indirect effect became insignificant in the sexual minority group (effect = –0.14, 95% boot CI –0.31 to 0.004) (Figure 3 and Table 4).

**Table 3:** The moderating effects of sexual orientation.

Outcome, Predictor		Coefficient	Boot SE	Boot LLCI^a^	Boot ULCI^b^
**Condom negotiation**					
	Sexual coercion	–0.43	0.14	–0.80	–0.24
	Sexual orientation	–1.96	0.77	–3.53	–0.50
	Inter 1^c^	0.36	0.15	0.16	0.74
**Condom use**					
	Sexual coercion	–0.29	0.68	–1.80	0.91
	Condom negotiation	1.87	0.39	–1.10	2.62
	Sexual orientation	–30.65	25.56	–78.20	23.27
	Inter 1	0.13	0.78	–1.26	1.77
	Inter 2^d^	0.04	0.90	–1.76	1.74

^a^LLCI: lower limit confidence interval.

^b^ULCI: upper limit confidence interval.

^c^Inter 1 = (sexual coercion) × (sexual orientation).

^d^Inter 2 = (condom negotiation) × (sexual orientation).

**Table 4:** Conditional indirect effects of the experience of sexual coercion on condom use.

Sexual orientation	Effect	Boot SE	Boot LLCI^a^	Boot ULCI^b^
Heterosexual	–0.80	0.33	–1.67	–0.36
Sexual minority	–0.14	0.10	–0.31	0.004

^a^LLCI: lower limit confidence interval.

^b^ULCI: upper limit confidence interval.

## Discussion

### Principal Findings

This study found a moderated mediation model of the pathway from sexual coercion to condom use via condom negotiation in a sample of Chinese female college students. Based on the Traumagenic Dynamics Model [20], the mediation effect of condom negotiation was tested and the results indicate that the relationship between sexual coercion and condom use is mediated by the level of condom negotiation. We found that a higher level of sexual coercion decreased condom negotiation, which in turn decreased condom use. The results were consistent with a previous study that was conducted on female sex workers from two West African countries [15].

In this study, sexual orientation moderated the indirect effect of sexual coercion on condom use. To our knowledge, this is the first known study to present such an intersectional analysis of the role of sexual orientation in the indirect effect of sexual coercion on condom use in young women. Altogether, these findings support the difference between heterosexual women and sexual minority women regarding the pattern of sexual behaviors in those who have experienced sexual coercion, emphasizing that sexual orientation meaningfully affects the relationship between sexual coercion and attitude toward condom negotiation. A significant indirect effect was found in the heterosexual women. This result is in line with a previous study in which the frequency of condom negotiation mediated the association between psychological intimate partner violence and condom use [35]. However, a nonsignificant indirect effect was found in sexual minority women, mainly because of the absence of condom negotiation. We also found no significant association between sexual coercion and condom negotiation or between condom negotiation and condom use in sexual minority women in this study. The belief that same-sex activities present a low risk for STIs is common in women who have sex with women (WSW) [36], and Formby [37] found that approximately 2 in 5 sexual minority women believe that they cannot get STIs from having sex with women. However, more recent research reported an infectious rate in WSW that was higher or similar to that in women who have sex exclusively with men [38,39]. The above mistaken belief mainly results in the absence of condom negotiation in WSW and their partners [40], which might contribute to the nonsignificant indirect effect in sexual minority women. Research by Walls [41] indicated that most sexual minority women seldom negotiate safe sex practices with their partners because they do not think they will contract STIs. This contributed to the nonsignificant indirect effect of condom negotiation on the relation between sexual coercion and condom use because condom negotiation might not be a critical factor affecting condom use in sexual minority women who have experienced sexual coercion. Instead of increasing condom negotiation skills, more information about the risks of STIs in sexual minority women and about appropriate protection methods should be provided.

### Study Limitations

Our findings should be interpreted with caution. One limitation is that our measurements relied on self-reports of sensitive information and often stigmatized experiences and behaviors, even though an anonymous process was used to minimize social desirability bias. Self-reports of sensitive information, such as sexual coercion experiences, are vulnerable to cognitive and motivational processes that can bias recall-based responses [42]. The other limitation is that a small sample of sexual minority women was included in the data analysis. Small sample sizes are a common problem in the same-sex sexual violence research field [43]. Larger sample sizes of sexual minority women with regional and religious diversity are needed to increase the statistical power.

### Future Work

Despite the above limitations, our study provided some new insights and implications for future studies examining condom use in women. One potential avenue for future research is to improve condom negotiation among sexual coercion survivors, given that the indirect effect of sexual negotiation accounted for nearly one-half (49.2%) of the total effect of sexual coercion on the consistency of condom use. The other implication is related to the different needs of women with different sexual orientations. In previous research, the difference in the mechanism of the relation between sexual coercion and sexual behaviors in heterosexual women and sexual minority women was ignored, and these two groups were included in the same intervention when addressing sexual coercion [44]. Our findings suggest that future interventions should not simply combine heterosexual women and sexual minority women. More qualitative and quantitative research to determine how sexual coercion experiences affect behavior changes in sexual minority women should be conducted.

### Conclusions

Condom negotiation was found to mediate the association between sexual coercion and condom use in young women. A further moderated mediation emerged, with the indirect effects of sexual coercion on the consistency of condom use via condom negotiation differing between sexual minority women and heterosexual women. The results emphasized that sexual orientation meaningfully affects the relationship between sexual coercion and condom negotiation, and the different patterns for individuals of different sexual orientations should be considered for future research and for interventions designed to mitigate the adverse effects of sexual coercion.

## Abbreviations

ICBI: interactive computer-based intervention
STI: sexually transmitted infection
WSW: women who have sex with women
